# Relationship between Cardiometabolic Parameters and Elevated Resting and Effort Heart Rate in Schoolchildren

**DOI:** 10.5935/abc.20170103

**Published:** 2017-09

**Authors:** Cristiane Fernanda da Silva, Miria Suzana Burgos, Priscila Tatiana da Silva, Leandro Tibiriçá Burgos, Letícia Welser, Ana Paula Sehn, Jorge André Horta, Elza Daniel de Mello, Cézane Priscila Reuter

**Affiliations:** 1 Universidade de Santa Cruz do Sul (UNISC), Santa Cruz do Sul, RS - Brazil; 2 Universidade Federal do Rio Grande do Sul, Porto Alegre, RS - Brazil

**Keywords:** Child Health, Adolescent Health, Metabolism Syndrome, Heart Rate, Physical Exertion, Rest

## Abstract

**Background:**

Little has been studied on heart rate and its relationship with metabolic disorders.

**Objective:**

To identify possible association between heart rate (HR) and metabolic disorders in children and adolescents.

**Methods:**

This cross-sectional study evaluated 2.098 subjects, aged between 7 and 17 years. The variables evaluated were: HR, systolic (SBP) and diastolic blood pressure (DBP), pulse pressure (PP), double-product (DP), myocardial oxygen consumption (mVO_2_), lipids, glucose and uric acid levels, body mass index (BMI) and waist circumference (WC). The values of HR at rest and effort were divided into quartiles. The association between continuous values of HR and cardiometabolic indicators was tested by linear regression.

**Results:**

LDL cholesterol presented a significantly higher mean (p = 0.003) in schoolchildren with resting HR greater or equal to 91 bpm, compared to students with less than 75 bpm. Compared with the quartiles of effort HR, SBP, DBP, glucose and uric acid presented high values when HR was greater or equal than 185 bpm. SBP, glucose and HDL cholesterol demonstrated a significant association with resting HR. Uric acid was observed as a predictor of increased effort HR.

**Conclusion:**

Schoolchildren with a higher resting HR have higher mean of LDL cholesterol. For effort HR, there was an increase in blood pressure, glucose and uric acid levels. Uric acid has been shown to be a predictor of elevated effort HR.

## Introduction

The search for new information that may contribute to the development of mechanisms for the prevention and treatment of cardiovascular and metabolic complications leads to different lines of research.^1^ In childhood and adolescence, the focus of these studies has been the association between obesity,^2^ cardiorespiratory fitness,^3^ changes in the lipid profile^4^ and non-communicable chronic diseases, such as diabetes and hypertension.^5^ However, a variable that has received increasing attention is cardiac autonomic modulation in children and adolescents.^1,6^

Cardiac autonomic changes can be investigated by means of a change in heart rate (HR).^7^ HR is a physiological variable that is easy to obtain and measure.^8^ Because it is a parameter of low cost and associated with reliable variables of effort measurement, HR is often used to assess cardiovascular system response during exercise and recovery.^9^ The association of HR with metabolic disorders has still been poorly studied, however, it is known that physical condition and the presence of pathologies may influence resting HR.^10^

A study carried out in Campinas-SP aimed to verify the existence of differences in the cardiovascular condition of obese and non-obese children under resting conditions. That study found that childhood obesity causes a greater overload on the resting heart due to a significant elevation of HR in obese children.^11^ According to Freitas Junior et al.^12^, a high amount of body fat leads to the release of inflammatory adipokines in the bloodstream which contributes to the development of chronic diseases, as well as the change in sympathetic and parasympathetic activities in children and adolescents, aspect which may cause an increase in resting heart rate.

Little has been studied about HR and its relationships with metabolic dysfunctions in childhood and adolescence.^8^ At rest, low heart rate values may be associated with health, reflecting a lower risk of cardiovascular diseases.^13^ A study by Fernandes et al.^14^ analyzed the association between resting HR and blood pressure in male children and adolescents and identified a positive association between these variables, suggesting that elevated HR also causes an increase in blood pressure in the pediatric population. Bruneto et al.^6^ have associated an increased morbidity rate and the onset of cardiovascular disease with the reduced practice of physical activity and low levels of conditioning in obese children and adolescents. In view of these perspectives, it is useful to investigate associations between heart rate and cardiometabolic indicators. In this way, we will be contributing to the construction of subsidies for the creation and implementation of health prevention and promotion policies aimed at improving the quality of life of the child and adolescent population. Thus, the present study aims to verify if there is an association between heart rate and metabolic dysfunctions in children and adolescents.

## Methods

The participants of this cross-sectional study are 2.098 subjects, female and male, aged between 7 and 17 years, belonging to municipal, state and private schools in the urban and rural area of the municipality of Santa Cruz do Sul, Rio Grande do Sul. The present study is part of a larger study entitled "Health of schoolchildren - Phase III", approved by the Committee of Ethics in Research (CEP) with Human Beings under protocol number 714.216 and CAAE: 31576714.6.0000.5343.

The variables used to evaluate this study were: resting and effort heart rate (HR), resting blood pressure (BP), pulse pressure (PP), double product (DP), myocardial oxygen consumption (mVO_2_), biochemical indicators, evaluated by means of serum (lipid and glycemic profile), anthropometrics: body mass index (BMI) and waist circumference (WC). To measure the resting HR, the student should be seated, with 5 minutes at rest. HR was assessed using the FT1 model frequency meter (Polar, Finland), with the heart rate sensor attached on the pectoral line over the sternum with an elastic band. The lowest value stabilized by the frequency meter was considered for resting HR. The exercise heart rate was evaluated after performing the 6-minute run/walk test, applied on the athletics track at the University of Santa Cruz do Sul-UNISC. For the 6-minute run/walk test, the guidelines of the protocol recommended by the Brazilian Sport Project - PROESP-BR^15^ were followed. The schoolchildren were previously instructed to wear light clothing and appropriate footwear (sneakers) and walk the longest distance possible throughout the test. The obtained results were collected immediately after the interruption of the test. Stress HR values were obtained through the frequency meter and expressed in beats per minute (bpm). For the evaluation of BP, the same procedure was applied to the resting HR, and the evaluation was performed in the left arm, with the student sitting. After 5 minutes at rest, two BP measurements were performed, using a sphygmomanometer, stethoscope and cuff suitable for the student's brachial perimeter. Only the lowest values of systolic (SBP) and diastolic (DBP) pressure were considered, and PP was obtained by the difference between SBP and DBP.^16^ All evaluations were performed at the University.

The biochemical indicators evaluated included lipid profile and glucose and uric acid levels, through serum samples from the children, who were oriented to maintain a previous fast of 12 hours. For the lipid profile, the following markers were measured: total cholesterol (TC) and HDL fraction (HDL-C) (high-density lipoprotein) as well as triglycerides. LDL-C (LDL-C) cholesterol was calculated according to the Friedewald, Levy and Fredrickson equation.^17^ Data obtained follow the recommendations of the National Heart, Lung, and Blood Institute^18^ and the American Diabetes Association,^19^ to assess the lipid and glycemic profile, respectively. All analysis were performed on automated equipment Miura One (I.S.E., Rome, Italy), using commercial kits.

BMI was obtained after weight and height were evaluated, using a balance and stadiometer, respectively. The BMI was calculated by dividing the weight by height, squared.^20^ The WC was evaluated with an inelastic tape measure, and the smallest perimeter of the trunk between the ribs and the iliac crest was evaluated.^21^ The values of DP were obtained by the calculation of the SBP x HR. The mVO_2_ values were obtained using the DP conversion formula: mVO_2_ = (DP x 0.0014) - 6.3, as proposed by Hellerstein and Wenger.^22^ The evaluation of the maturational stage was performed by Tanner's classification, considering the maturational development in 5 phases, for both sexes, being evaluated the development of pubic hairiness and genitalia. For the application of the test, each of the phases was shown to the student through drawings and he was oriented to choose the phase that most closely resembled his current stage of development.^23^

### Statistical analysis

Statistical analysis of the data was performed in the statistical program SPSS v. 23.0 (IBM, Armonk, NY, USA). The descriptive characteristics were presented in frequency and percentage for categorical variables. For continuous variables, the Shapiro-Wilk test was used to test the normality of the data (HR at rest and effort, SBP, DBP, PP, glucose, TC, HDL-C, LDL-C, TG and uric acid). Data were presented on average (standard deviation), since they had a normal distribution. Subsequently, HR values of rest and effort were divided into quartiles. Comparison of the mean values of the cardiometabolic indicators, according to categorization with the resting HR quartiles (Q1: < 75 bpm, Q2: 75-82 bpm, Q3: 83-90 bpm and Q4: ≥ 91 bpm) and effort (Q1: < 152 bpm; Q2: 152-171 bpm, Q3: 172-184 bpm and Q4: ≥ 185 bpm), was performed using analysis of variance (ANOVA), with a Tukey Post Hoc test for comparison between groups. The association between the continuous values of rest and effort HR with the cardiometabolic indicators was tested by linear regression, adjusted for the variables gender, age, body mass index and maturational stage. For all analysis, the differences for p <0.05 were considered significant.

## Results

The characteristics of the evaluated students, with respect to sex, maturational stage, age and rest and effort HR, can be visualized in table 1. Of the 2.098 students evaluated, 903 (43%) were males and 1195 (57%) female, with a mean age of 11.50 ± 2.77 years.

**Table 1:** Descriptive characteristics of subjects. Santa Cruz do Sul, RS, 2014-2015

	n (%)
**Sex**	
Male	903 (43)
Female	1195 (57)
**Maturational stage**	
I	517 (25)
II	510 (24)
III	437 (21)
IV	478 (23)
V	156 (7)
	**Mean (standard deviation)**
Age (years)	11.50 (2.77)
Resting HR(bpm)	82.67 (10.40)
Effort HR (bpm)	168.40 (22.47)

Comparison of the mean values of cardiometabolic indicators, according to resting HR quartiles (Table 2), shows that there was a significant association between DP (0.678 p < 0.001) and mVO_2_ (0.678 p < 0.001) with HR, in rest. For LDL-C values, there was also a significant difference (p = 0.003), with a higher mean in the fourth quartile (88.68 mg / dL) compared to the first quartile (82.66 mg/dL). In addition, a significant difference was observed in the values of DP and mVO_2_ from one quartile to another and all quartiles differed from each other. There was no significant association between PP and HR.

**Table 2:** Comparison of mean values of cardiometabolic indicators according to resting HR quartiles

	Quartiles of resting HR				p
	**Q1 (n = 513)**	**Q2 (n = 515)**	**Q3 (n = 540)**	**Q4 (n = 530)**	
	**< 75 bpm**	**75-82 bpm**	**83-90 bpm**	**≥ 91 bpm**	
SBP (mmHg)	106.94 (14.61)	105.95 (13.85)	105.03 (14.28)	107.02 (14.94)	0.081
DBP (mmHg)	65.47 (10.60)	64.62 (10.75)	64.49 (11,01)	65.18 (11.19)	0.412
Glucose (mg/dL)	89.85 (9.52)	89.44 (9.29)	89.31 (9.49)	90.24 (9.14)	0.354
TC (mg/dL)	158.62 (30.93)	160.60 (30.20)	161.85 (30.32)	163.70 (31.22)	0.055
HDL-C (mg/dL)	62.16 (10.86)	60.95 (11.48)	60.77 (12.40)	60.49 (11.30)	0.098
LDL-C (mg/dL)	82.66 (28.45)	85.11 (25.93)	86.47 (26.03)	88.68 (26.61)	0.003^I^
TG (mg/dL)	69.82 (30.76)	71.80 (31.61)	72.75 (36.96)	71.73 (34.40)	0.552
UA (mg/dL)	4.30 (1.57)	4.14 (1.26)	4.16 (2.44)	4.16 (1.19)	0.381
DP (bpm/mmHg)	7354.33 (1073.25)	8364.62 (1136.90)	9087.20 (1253.50)	10257.53 (1522.47)	< 0.001^II^
mVO_2_ (mlO_2_/100.LV.min)	3.99 (1.50)	5.41 (1.59)	6.42 (1.75)	8.06 (2.13)	< 0.001^II^
PP (mmHg)	41.47 (9.69)	41.34 (9.22)	40.54 (9.56)	41.84 (10.68)	0.176

Analysis of variance (ANOVA); Data expressed as mean (standard deviation); Q1: quartile 1; Q2: quartile 2; Q3: quartile 3; Q4: quartile 4; Bpm: beats per minute; SBP: systolic blood pressure; DBP: diastolic blood pressure; TC: total cholesterol; HDL-C: high-density lipoprotein; LDL-C: low-density lipoprotein; TG: triglycerides; UA: uric acid; DP: double-product; MVO_2_: consumption of oxygen by the myocardium; PP: pulse pressure; Tukey Post Hoc:

I Significant difference from Q1 to Q4 (p = 0.002);

II significant difference from Q1 to Q2 (p < 0.001), for Q3 (p < 0.001) and for Q4 (p < 0.001); (p < 0.001), from Q2 to Q4 (p < 0.001) and from Q3 to Q4 (p < 0.001).

When the cardiometabolic indicators were compared with the effort HR quartiles (Table 3), mean values were higher in the fourth quartile compared to the first quartile for SBP (108.71 mmHg); p < 0.001), DBP (66.43 mmHg, p < 0.001), glucose (90.58 mg/dL, p = 0.028) and uric acid (4.40 mg/dL, p < 0.001).

**Table 3:** Comparison of the mean values of the cardiometabolic indicators according to the quartiles of the effort HR

	Quartiles of effort FC				p
	Q1 (n = 508)	Q2 (n = 537)	Q3 (n = 506)	Q4 (n = 547)	
	< 152 bpm	152-171 bpm	172-184 bpm	≥ 185 bpm	
SBP (mmHg)	103.72 (13.80)	105.44 (13.83)	106.89 (14.69)	108.71 (14.95)	< 0.001^I^
DBP (mmHg)	63.52 (10.59)	64.25 (10.28)	65.47 (10.81)	66.43 (11.63)	< 0.001^II^
Glucose (mg/dL)	89.23 (9.90)	89.93 (9.40)	89.02 (8.72)	90.58 (9.34)	0.028^III^
TC (mg/dL)	159,21 (30.31)	161.47 (27.42)	162.73 (33.54)	161.45 (31.34)	0.323
HDL-C (mg/dL)	60.80 (11.86)	61.42 (11.16)	60.62 (11.54)	61.45 (11.63)	0.544
LDL-C (mg/dL)	84.12 (26.79)	85.84 (24.29)	87.80 (28.78)	85.33 (27.33)	0.175
TG (mg/dL)	70.82 (31.96)	70.25 (31.88)	72.25 (36.33)	72.83 (34.00)	0.559
Uric acid (mg/dL)	3.91 (1.20)	4.28 (2.33)	4.16 (1.21)	4.40 (1.70)	< 0.001^IV^

Analysis of variance (ANOVA); Data expressed as mean (standard deviation); Q1: quartile 1; Q2: quartile 2; Q3: quartile 3; Q4: quartile 4; Bpm: beats per minute; SBP: systolic blood pressure; DBP: diastolic blood pressure; HDL-C: high-density lipoprotein; LDL-C: low density lipoprotein; Post Hoc of Tukey:

I Significant difference of Q1 for Q3 (p = 0.002) and Q1 for Q4 (p < 0.001);

II significant difference from Q1 to Q3 (p = 0.022) and Q1 to Q4 (p < 0.001);

III significant difference from Q3 to Q4 (p = 0.035);

IV significant difference from Q1 to Q2 (p = 0.002) and Q1 to Q4 (p < 0.001).

When the association between HR and cardiometabolic indicators is analyzed by means of linear regression (Table 4), SBP, glucose and HDL-C were associated with resting HR; however, this association, although significant, was weak. An association between resting HR and mVO_2_ (β = 3.46; p < 0.001) was found. For exercise HR, only uric acid was associated (β = 0.73, p = 0.015), demonstrating that it was a predictor of increased exercise HR in the sample evaluated. On the other hand, when the Pearson correlation coefficient was evaluated, a moderate association was found only between resting HR with DP (r = 0.678, p < 0.001) and mVO_2_ (r = 0.678, p < 0.001).

**Table 4:** Association between heart rate and cardiometabolic indicators Rest HR Effort HR

	Rest HR					Effort HR				
	**β**	**SE**	**p^1^**	**r**	**p^2^**	**β **	**EP**	**p^1^**	**r**	**p^2^**
SBP	0.08	0.02	0.001	-0.00	0.967	0.08	0.05	0.096	0.13	< 0.001
DBP	0.02	0.03	0.500	-0.01	0.795	0.01	0.07	0.884	0.11	< 0.001
Glucose	0.05	0.02	0.034	0.01	0.684	0.01	0.05	0.911	0.05	0.030
Total cholesterol	0.16	0.08	0.060	0.07	0.001	-0.22	0.19	0.252	0.04	0.100
HDL-C	-0.20	0.09	0.022	-0.05	0.019	0.27	0.19	0.156	0.02	0.451
LDL-C	-0.14	0.08	0.096	0.09	< 0.001	0.26	0.19	0.168	0.03	0.122
Triglycerides	-0.02	0.02	0.285	0.04	0.062	0.02	0.04	0.631	0.01	0.546
Uric acid	-0.03	0,13	0.806	-0.03	0.132	0.73	0.30	0.015	0.09	< 0.001
DP	0.01	0.00	< 0.001	0.678	< 0.001	-	-	-	-	-
mVO_2_	3.46	0.07	< 0.001	0.678	< 0.001	-	-	-	-	-
PP	0.07	0.02	0.003	0.005	0.820	-	-	-	-	-

Linear regression adjusted for sex, age, body mass index and maturational stage; SE: standard error; R: Pearson's correlation;

1 value of significance for the linear regression test;

2 significance value for the Pearson correlation test; HR: heart rate; SBP: systolic blood pressure; DBP: diastolic blood pressure; HDL-C: high-density lipoprotein; LDL-C: low-density lipoprotein; DP: double-product; MVO_2_: consumption of oxygen by the myocardium; PP: pulse pressure.

## Discussion

The present study sought to evaluate possible associations between heart rate and metabolic disorders. We found that high resting HR (equal to or greater than 91 bpm) was associated with higher LDL-C levels (88.68 mg/dL, p < 0.001). High HR was associated with high SBP values (108.71 mmHg, p < 0.001), DBP (66.43 mmHg, p < 0.001), glucose (90.58 mg/dL, p = 0.028) and uric acid (4.40 mg/dL, p < 0.001). A study in Presidente Prudente-SP found similar data and showed a positive association between resting HR and dyslipidemia, and students with higher values of HR had higher levels of TC and triglycerides. However, no association was found with LDL-C values.^12^

In our study, the analysis of the association between HR and cardiometabolic indicators found a significant association among SBP, glucose and HDL-C, with resting HR, but this association was weak. Only uric acid, related to exercise HR, showed a good association (β = 0.73, p = 0.015), which may be considered a predictor of increased cardiometabolic risks in the sample studied, considering that uric acid at high levels is associated with the occurrence of MS in adolescents.^24,25^ The mechanism by which the relationship between uric acid and MS could be explained is in the fact that the hepatic consequence of MS is expressed through non-alcoholic fatty liver disease (NAFLD) .^26^

Thus, alterations in some specific components of MS, such as BP, glucose metabolism and lipids, suggest to be indicators of the aggravating installation of MS, including initial manifestations of NAFLD, an aspect that in this study already seems to have an impact on lower cardiovascular conditions for activities with high respiratory demands, thus reflecting a change in effort HR values. Likewise, uric acid, as an isolated component, would also be a more sensitive variable to capture these alterations. This hypothesis would be justified by the finding of NAFLD in the evaluated students. However, this relationship was not tested in the present study. On the other hand, a study carried out in Campina Grande, Brazil, with 129 children and adolescents aged 2 to 18 years, evaluated the relationship between uric acid concentration according to the presence of NAFLD and / or MS in children and adolescents with excess weight. The study identified that high levels of uric acid are associated with MS, SBP and adolescence, but this association was not observed with NAFLD.^27^

However, the low age range used in the study might have hampered the finding of NAFLD, since the condition would take time to set up. There is evidence that uric acid levels are significantly lower in children than in adolescents (4.74 ± 1.05 vs. 5.52 ± 1.49 mg/dL, p < 0.001), with boys tending to reach higher peak of uric acid between 12-14 years of age and girls between 10-12 years.^28^ In addition, the study pointed out that individuals with elevated SBP were four times more likely to have hyperuricemia and that high levels of uric acid were associated with individual components of MS, such as BMI, WC and BP, which is similar to the findings pointed out in this study.^27^

Likewise, although the association of SBP, glucose and HDL-C with HR was weak, these data suggest to be indicative of the establishment of an early stage of MS, considering that recent studies have indicated positive associations between MS and changes in these metabolic indicators.^29,30^ Recent data from the Cardiovascular Risk Study in Adolescents-ERICA, performed with 37.504 Brazilian adolescents from 27 capitals, showed that the combinations between high BP, high triglycerides and low HDL-C are the most frequently responsible for the diagnosis of MS in schoolchildren.^29^ Thus, the data presented in this research point to elevated levels of HR as independent factor for the risk of dyslipidemias and cardiovascular disorders.^31,32^

On the other hand, a study carried out in 27 European cities evaluated 769 adolescents to verify the ability of HR to screen for metabolic risk factors and found that resting HR is not a good predictor for cardiometabolic risks. According to the study, the resting HR provides an underestimated value, so positive associations in studies between chronic degenerative diseases and resting HR would have been found due to the use of the percentiles scale, which would be a limited parameter, as it did not present the accuracy and precision of the HR rates; Thus, the study, using a better analysis, called the ROC curve, could consider the results presented by the study as more reliable. However, the same study pointed out that the isolated analysis of the risk factors identified that the male adolescents presented higher values of SBP (124.4 mmHg, 95% CI: 123.1-125.8) and TC/HDL (3.02; 95% CI: 2.96-3.09) compared to female adolescents (BP: 116.2 mmHg, 95% CI: 115.3-117.1, TC/HDL 2.99; 95% CI: 2.93-3.04). In addition, when compared with their female counterparts, the boys also presented higher resting HR, an aspect that suggests that higher resting HR levels are associated with an increase in BP and TC/HDL.^31^

In addition, changes in the behavior of HR of adolescents seem to be related to MS, both regarding changes to higher values and to reduced values. Studies with adolescents with obesity and alterations in the metabolic profile have shown that those with higher glucose levels, levels of TG, LDL-C and BP associated with obesity, in response to increased insulin resistance, alter the functions sympathetic and parasympathetic and results in a decrease in cardiac function and perhaps a reduction in HR values. This long-term fact would explain, in part, the increased risk for cardiovascular events and sudden death in obese individuals.^33,34^

Positive associations between resting HR and cardiovascular risk factors in adolescents were also found in a study carried out in 48 municipalities in the state of Pernambuco, (Northeastern Brazil), in which 4619 adolescents aged 14 to 19 years were evaluated. The study evaluated a set of risk factors and found that resting HR was associated with abdominal obesity (b = 0.106, p = 0.003), sedentary behavior (b = 0.099, p < 0.001), high BP (b = 0.160; p < 0.001) and physical inactivity (b = 0.049, p = 0.034) in boys, and in girls showed association with high BP (b = 0.259, p < 0.001). Moreover, the presence of five risk factors in schoolchildren resulted in significantly higher values of resting HR (p < 0.05), compared to schoolchildren with no cardiovascular risk factors. Thus, study data suggested that resting HR at or above 82.5 bpm (± 13.9 bpm) in boys and 89.8 bpm (± 10.9 bpm) in girls could be considered as a risk factor for CVD.^35^

In relation to the other cardiometabolic components evaluated, our study found a significant association between DP (0.678, p < 0.001) and mVO_2_ (0.678, p < 0.001) with HR at rest. In addition, the comparison of mean values of cardiometabolic indicators, according to the resting HR quartiles, showed a significant difference in the values of DP and mVO_2_ from one quartile to another and all quartiles differed from each other. These findings provide new perspectives for the use and approach of resting HR, since it is known that elevated HR values at rest are associated with worse functional conditions at more advanced ages.^36^ However, there is scarce information available in the literature for analysis of these variables in the child and adolescent population. Due to the fact that both DP and mVO_2_ express the conditions and demands of cardiac work, it is assumed that for the population evaluated, high resting HR can be considered as indicative of the occurrence of health problems or other compromises related to cardiac function, considering the high level of effort expended by the myocardium to develop the vital functions at rest.^37^

Thus, the hypothesis raised by the data found in our study indicates that the higher the resting HR, the greater the cardiac overload of the students. Therefore, elevated HR could be used as a predictor, in the first analysis, of altered physiological responses in children and adolescents, and the use of DP and mVO_2_ values contributes to a better vision of the cardiac outcome. Moreover, associations among HR, obesity and cardiometabolic variables have indicated that overweight in children and adolescents cause greater impairment in cardiorespiratory fitness and pulmonary function, both at maximum and submaximal level, when compared to adolescents with normal weight, mainly By the commitment of oxygen consumption.^38,39^

Thus, in providing data demonstrating homeostatic changes in the child and adolescent population, our study supports the evidence that elevated cardiac responses indicate greater physiological fragility. Likewise, this study provides support for the development of new research aimed at better investigating this association, considering that no data were found in the literature regarding studies on possible interactions among HR, DP and mVO_2_ with metabolic variables in children and adolescents.

At the same time, it should be noted that this study, however, has some limitations. The infeasibility of evaluating DP and mVO_2_ values of effort prevents a broader view of cardiac function and the approach of this variable for the population surveyed. Simultaneously, the unavailability of studies relating such components to this public, restricts the understanding of the data and the formulation of hypotheses. Likewise, the lack of reference values for DP and mVO_2_ variables for the pediatric population makes it difficult to interpret the results. However, the analysis presented here were adjusted to the characteristics of the study population and the associations found deserve to be further explored in future studies related to the health of schoolchildren, considering that changes in resting HR, SBP and DP, similar to those found in our analysis, have also been predictive of mortality from coronary events and increased risk of functional decline over the years.^35,40^ DP is also known to be a stronger predictor of cardiac events than PA, HR and mVO_2_, an aspect that demonstrates the importance of including these variables in the analysis of cardiac function.^40^

In this sense, the data found in this study can be seen as a small cut of an association that needs to be further investigated, since HR indicates to be a potential measure for the diagnosis of metabolic and cardiovascular diseases. However, future research is needed to determine whether these measures may be useful for screening for physiological changes related to HR dynamics in children and adolescents.

## Final considerations

Schoolchildren with resting heart rate equal to or greater than 91 beats per minute present higher mean LDL cholesterol. For exercise heart rate, schoolchildren with 185 or more beats per minute had elevated systolic and diastolic blood pressure and glucose and uric acid levels. In addition, uric acid has been shown to be a predictor of elevated effort heart rate.
